# An Evaluation of Community Assessment Tools (CATs) in Predicting Use of Clinical Interventions and Severe Outcomes during the A(H1N1)pdm09 Pandemic

**DOI:** 10.1371/journal.pone.0075384

**Published:** 2013-09-19

**Authors:** Malcolm G. Semple, Puja R. Myles, Karl G. Nicholson, Wei Shen Lim, Robert C. Read, Bruce L. Taylor, Stephen J. Brett, Peter J. M. Openshaw, Joanne E. Enstone, James McMenamin, Barbara Bannister, Jonathan S. Nguyen-Van-Tam

**Affiliations:** 1 Institute of Translational Medicine, University of Liverpool, Liverpool, United Kingdom; 2 Division of Epidemiology and Public Health, University of Nottingham, Nottingham, United Kingdom; 3 Infectious Diseases Unit, University Hospitals of Leicester NHS Trust, Leicester Royal Infirmary, Leicester, United Kingdom; 4 Department of Respiratory Medicine, Nottingham University Hospitals NHS Trust, Nottingham, United Kingdom; 5 Clinical and Experimental Sciences, University of Southampton, Southampton General Hospital, Southampton, United Kingdom; 6 Department of Critical Care, Portsmouth Hospitals NHS Trust, Portsmouth, United Kingdom; 7 Centre for Peri-operative Medicine and Critical Care Research, Imperial College Healthcare NHS Trust, London, United Kingdom; 8 Centre for Respiratory Infections, National Heart and Lung Institute, Imperial College, London, United Kingdom; 9 Health Protection Scotland, NHS National Services, Glasgow, United Kingdom; 10 Infectious Diseases Department, Royal Free Hampstead NHS Trust, London, United Kingdom; University of Hong Kong, Hong Kong

## Abstract

During severe influenza pandemics healthcare demand can exceed clinical capacity to provide normal standards of care. Community Assessment Tools (CATs) could provide a framework for triage decisions for hospital referral and admission. CATs have been developed based on evidence that supports the recognition of severe influenza and pneumonia in the community (including resource limited settings) for adults, children and infants, and serious feverish illness in children. CATs use six objective criteria and one subjective criterion, any one or more of which should prompt urgent referral and admission to hospital. A retrospective evaluation of the ability of CATs to predict use of hospital-based interventions and patient outcomes in a pandemic was made using the first recorded routine clinical assessment on or shortly after admission from 1520 unselected patients (800 female, 480 children <16 years) admitted with PCR confirmed A(H1N1)pdm09 infection (the FLU-CIN cohort). Outcome measures included: any use of supplemental oxygen; mechanical ventilation; intravenous antibiotics; length of stay; intensive or high dependency care; death; and “severe outcome” (combined: use of intensive or high dependency care or death during admission). Unadjusted and multivariable analyses were conducted for children (age <16 years) and adults. Each CATs criterion independently identified both use of clinical interventions that would in normal circumstances only be provided in hospital and patient outcome measures. “Peripheral oxygen saturation ≤92% breathing air, or being on oxygen” performed well in predicting use of resources and outcomes for both adults and children; supporting routine measurement of peripheral oxygen saturation when assessing severity of disease. In multivariable analyses the single subjective criterion in CATs “other cause for clinical concern” independently predicted death in children and in adults predicted length of stay, mechanical ventilation and “severe outcome”; supporting the role of clinical acumen as an important independent predictor of serious illness.

## Introduction

Healthcare demand during severe influenza pandemics can exceed clinical capacity to provide normal standards of care [1], a phenomenon now known as “surge”. National pandemic preparedness plans include contingencies for surge capacity including reducing non-urgent activity and expanding critical care facilities. [2], [3] In exceptional circumstances the use of triage is recommended to rationalise use of limited hospital resources. [3].

Assessment tools could provide a framework for consistent and rational triage decisions pertaining to hospital referral. Ideally, tools used in the community by a General Practitioner (GP)* should match hospital admission triage criteria, and be valid predictors of the likelihood to benefit from use of hospital-based interventions. [*The term General Practitioner is used in the UK and some other countries to indicate a registered medical practitioner specialised in providing acute and chronic care to people of all ages and sexes in the community and in other countries may be known as a usual community doctor or primary care physician.].

During peak pandemic activity, healthcare worker absence is potentially increased due to personal sickness and caring for relatives. Imaging and laboratory services may also be limited. Together these place increased reliance and emphasis on clinical history-taking and examination skills for triage decisions, which may be devolved to less experienced staff.

Provisional guidance [4] suggested the use of the CURB-65 pneumonia score [5] and the Pandemic Medical Early Warning Score (PMEWS) [6] for hospital triage of adults in the UK. Importantly, neither score was ever intended for use in children. Most children can benefit from access to adult critical care facilities and general intensive care units either if they are unlikely to need transfer to a paediatric intensive care unit (PICU), or if in extreme circumstances there is no PICU capacity.

Recognising this gap, a “tool-kit” of national guidance was developed in 2008 in the UK which included newly developed Community Assessment Tools (CATs) for both children (Appendix S1) and adults (Appendix S2) in primary and secondary care, and matched hospital care pathways. [3], [7] CATs were designed to help front-line staff identify sick children and adults most likely to benefit from hospital care during pandemic surge periods. Although not specific to influenza, they nevertheless address the most likely modes of critical illness arising from the disease or its complications. Criteria fields are common to adult and paediatric CATs, however specific physiological thresholds and definitions (e.g. respiratory rate) are age-appropriate.

CATs use six objective criteria and one subjective criterion, any one or more of which should prompt urgent referral and admission to hospital (see Appendices 1 & 2). The evidence base for the objective criteria is that which supports the recognition of severe influenza and severe pneumonia in the community (including resource-limited settings) for adults and children, severe chronic obstructive pulmonary disease in adults, serious feverish illness in children and severe bronchiolitis in infants. [5], [8]–[17] Evidence has recently been published supporting use of subjective criteria in children [18].

The concept was developed by a group at Alder Hey Children’s Hospital NHS Foundation Trust Liverpool UK (Appendix S3), as part of an integrated package of care, which included community and hospital pathways. The pathways provide safety measures for people who are moderately unwell or who may have other diagnoses but cannot be admitted to hospital at the time of triage. The CATs and pathways were further developed by paediatric and adult expert clinical development groups and published by the Department of Health (England) for use by the National Health Service. [3] Clinicians were, and are, warned not to use the CATs and the pathways unless the local situation precludes normal admission and discharge processes.

During the somewhat mild 2009–10 influenza pandemic in the UK, some local areas came under surge pressure, especially for critical care, but overall national clinical capacity was not exceeded and CATs and surge pathways were not implemented. Notwithstanding, data collected by the UK Influenza Clinical Information Network (FLU-CIN) cohort provided an opportunity for a retrospective evaluation of the predictive ability of CATs in a pandemic scenario.

## Methods

FLU-CIN was an emergency surveillance study established by the Department of Health England. The details of data collection and the findings have been described elsewhere. [19] In brief FLU-CIN used a purposive sampling frame based on 13 sentinel hospitals situated in five clinical ‘hubs’ in Nottingham, Leicester, London, Sheffield and Liverpool, with contributions from a further 45 non-sentinel hospitals in England and 17 in Scotland, Wales and Northern Ireland. Between April 2009 and January 2010, clinico-epidemiological and outcome data were collected on 1520 patients (800 female, 480 children <16 years) admitted to participating UK hospitals with A(H1N1)pdm09 infection, confirmed by reverse transcribed polymerase chain reaction (PCR) of respiratory samples obtained during the admission episode. Table 1 summarises socio-demographic characteristics of the FLU-CIN cohort. The clinical characteristics of the FLU-CIN cohort have been described elsewhere. [20] This was a case-control analysis using retrospective data from the full FLU-CIN cohort data. Criterion data were gathered from routine case notes using the first recorded routine clinical assessment on or shortly after admission.

**Table 1:** Demographic characteristics of 1520 UK patients hospitalised with pandemic H1N1 infection during the 2009/10 pandemic (The FLU-CIN Cohort) compared with source population and pre-pandemic hospital data on acute respiratory infection admissions.

Demographic characteristic		n (%)	UK Population, %	Pre-pandemic, acute respiratory infections*, %
Sex†	Male	720 (47.4)	48.7	50.4
	Female	800 (52.6)	51.3	49.6
Age (years)^‡^	<1	121 (8.0)	1.3	14.7
	1–4	138 (9.1)	4.8	12.6
	5–15	221 (14.5)	12.6	4.8
	16–24	245 (16.1)	12.1	2.5
	25–34	242 (15.9)	12.9	3.3
	35–44	195 (12.8)	14.6	4.6
	45–54	168 (11.0)	13.5	5.4
	55–64	115 (7.6)	11.8	7.9
	65–74	55 (3.6)	8.5	11.0
	>75	20 (1.3)	7.8	33.2
Ethnicity‡‡	White	630 (41.5)	92.1	83.3
	Mixed	11 (0.7)	1.2	1.9
	Asian/Asian British	249 (16.4)	4.0	8.7
	Black/Black British	129 (8.5)	2.0	3.4
	Chinese and other	121 (8.0)	0.8	2.7
	Ethnicity missing	380 (25.0)	–	–

Data are numbers (%), unless otherwise indicated.

† Census 2001 Data for comparison of sex (KS01 tables) were obtained from the Office for National Statistics (ONS) (www.statistics.gov.uk). ^‡^Demographic data on age distribution based on 2009 mid-year population estimates, ONS.

‡‡ Ethnicity data from ONS (Census 2001 data, 2001 data from the General Register Office for Scotland and Northern Ireland Statistics and Research Agency). *Hospital Episodes Statistics (HES) data: primary discharge codes relating to possible influenza admissions (J06, J10, J11, J13–22) were considered for the pre-pandemic influenza active period Nov 2008-Mar 2009.

We investigated the association between each CATs criterion (separately) and the use of hospital-based clinical interventions as well as patient outcomes. Next, we used multivariable logistic regression modelling to identify which of the CATs criteria were independent predictors of the use of clinical interventions and patient outcomes. All criteria were included in the multivariable model in the first instance irrespective of statistical significance. Thereafter, we used an automatic stepwise forward selection approach (threshold for selection p<0.05) to compare the resultant multivariable model with our original approach.

The CATs criteria are:

- severe respiratory distress,
- increased respiratory rate,
- oxygen saturation ≤92% on pulse oximetry breathing air, or “on oxygen”,
- respiratory exhaustion,
- evidence of severe clinical dehydration or clinical shock,
- altered consciousness level and
- causing other clinical concern to the patient’s GP or clinical team.

All criteria were validated using separately recorded clinical observations made by regular clinical staff on or shortly after admission.

Outcome measures included clinical interventions and patient outcomes: any use of supplemental oxygen; mechanical ventilation; intravenous antibiotics; length of stay (categorised as >48 hours; ≥6 days and ≥12 days); higher levels of care (high dependency – level 2 or intensive care – level 3*); death; and “severe outcome” defined by a composite of any use of higher levels of care (levels 2 or 3) or death during admission. [*Level 0: patients whose care needs can be met through normal ward care; level 1: patients at risk of deteriorating or recently relocated from higher levels of care whose needs can be met on an acute ward with additional advice and support from the critical care team; level 2: patients requiring more detailed observation or intervention, including support for a single failing organ system and those ‘stepping down’ from higher levels of care high dependency unit; level 3: patients requiring advanced respiratory support alone or basic respiratory support together with support of at least two organ systems. This includes all complex patients requiring support for multi-organ failure in intensive care units].

Analyses were conducted separately for children (age <16 years) and adults (≥16 years). Results are presented as unadjusted and adjusted odds ratio (OR) with 95% confidence intervals (95%CI) and p values. The OR describes the likelihood of a given outcome when an individual CAT criterion is positive. This was a secondary data analysis based on pragmatic recording of routine clinical assessments by regular clinical staff. The underpinning assumption is that the data recorded on presence of criteria was complete; however there is no way to verify this. By default, where a criterion was not recorded as being present, that criterion was assumed to be absent i.e. “If it isn’t in the notes it didn’t happen”. [21] Thus there are no missing data in the analysis. All analyses were carried out using Stata version 11.0 (StataCorp. 2009. Stata Statistical Software: Release 11.0; Stata Corporation, College Station, TX, USA).

### Ethics Statement

The Ethics and Confidentiality Committee of the National Information Governance Board for Health and Social Care in England approved FLU-CIN procedures before commencement of the study. The collection of data for the FLU-CIN database has been publicly funded and, as a public good, will be made available for new research purposes on a case-by-case basis. In general, only anonymised data will be supplied to researchers, except where the law permits the processing of identifiable data. Ownership and oversight of data access and use resides with the Pandemic Influenza Preparedness Team at the Department of Health, England. Any requests for access to FLU-CIN data should be made to Department of Health via the project leader, Prof JS Nguyen-Van-Tam (jvt@nottingham.ac.uk).

## Results

The unadjusted analysis investigating the association between each CATs criterion at admission (separately) and outcome measures (hospital-based intervention and patient outcomes) are presented for children (<16 years) in Table 2 and for adults (≥16 years) in Table 3. The distribution of study subjects meeting each criterion, tabulated by every outcome measure is reported in Table S1.

**Table 2:** Unadjusted analysis investigating the association between meeting CAT criteria and outcome measures in children (<16 years).

Outcome measure	A: Severe respiratorydistress	B: Increasedrespiratory rate	C: Oxygen saturation≤92% in air or onoxygen	D: Respiratoryexhaustion	E: Severe clinicaldehydration or shock	F: Alteredconsciousness	G: Other clinicalconcern
Supplemental oxygen	**5.28 (3.34–8.34),** *<0.001*	**1.59 (1.03–2.45),** *0.035*	X	*	1.71 (0.31–9.49), *0.537*	**2.53 (1.39–4.60),** *0.002*	1.88 (0.95–3.72), *0.071*
Mechanical ventilation	**2.82 (1.61–4.93),** *<0.001*	1.08 (0.61–1.89), *0.798*	**5.53 (3.09–9.87),** *<0.001*	*	**7.62 (1.50–38.7),** *0.014*	**5.56 (2.90–10.7),** *<0.001*	0.78 (0.35–1.77), *0.555*
IV antibiotics	1.46 (0.82–2.60), *0.204*	**0.32 (0.19–0.52),** *<0.001*	**3.66 (1.77–7.57),** *<0.001*	*	0.38 (0.07–2.12), *0.271*	1.10 (0.50–2.44), *0.809*	**2.63 (1.21–5.69),** *0.014*
Length of stay >48 hours	**2.24 (1.34–3.73),** *0.002*	1.40 (0.90–2.17), *0.134*	**4.46 (2.52–7.90),** *<0.001*	*	0.66 (0.11–3.99), *0.650*	1.33 (0.67–2.65), *0.422*	1.21 (0.57–2.58), *0.618*
Length of stay ≥6 days	**1.73 (1.13–2.63),** *0.011*	**2.12 (1.43–3.16),** *<0.001*	**1.97 (1.30–2.98),** *0.001*	*	4.68 (0.85–25.8), *0.077*	1.20 (0.66–2.20), *0.552*	1.07 (0.54–2.13), *0.851*
Length of stay ≥12 days	1.33 (0.81–2.19), *0.256*	**2.22 (1.39–3.54),** *0.001*	1.16 (0.70–1.91), *0.564*	*	2.22 (0.40–12.3), *0.360*	1.33 (0.67–2.65), *0.417*	1.26 (0.58–2.75), *0.558*
Mortality	**3.45 (1.33–8.93),** *0.011*	2.09 (0.81–5.39), *0.128*	1.59 (0.60–4.19), *0.348*	*	**14.3 (2.44–84.0),** *0.003*	**3.32 (1.13–9.71),** *0.029*	**4.55 (1.54–13.5),** *0.006*
Severe outcomes (level 2/3 admission or death)	**3.16 (1.91–5.22),** *<0.001*	1.53 (0.94–2.50), *0.090*	**4.95 (2.97–8.25),** *<0.001*	*	**11.0 (1.98–61.1),** *0.006*	**6.44 (3.49–11.9),** *<0.001*	**2.38 (1.16–4.90),** *0.019*

Values are unadjusted odds ratios (95% confidence intervals), significant values (p<0.05) in bold and p values in italic. X Not calculated as this criterion typically directs the outcome (use of supplemental oxygen). *Not possible to calculate odds ratios.

**Table 3:** Unadjusted analysis investigating the association between meeting CAT criteria and outcome measures in adults (≥16 years).

Outcome measure	A: Severe respiratorydistress	B: Increasedrespiratory rate	C: Oxygen saturation≤92% in air or onoxygen	D: Respiratoryexhaustion	E: Severe clinicaldehydration or shock	F: Alteredconsciousness	G: Other clinicalconcern
Supplemental oxygen	**5.01 (3.73–6.74),** *<0.001*	**1.46 (1.08–1.97),** *0.013*	X	**4.26 (1.79–10.1),** *0.001*	**1.48 (1.06–2.07),** *0.023*	**2.29 (1.31–4.02),** *0.004*	0.71 (0.44–1.14), *0.152*
Mechanical ventilation	**2.41 (1.63–3.58),** *<0.001*	**2.92 (1.99–4.28),** *<0.001*	**8.25 (5.22–13.0),** *<0.001*	**10.0 (4.29–23.3),** *<0.001*	**2.42 (1.59–3.67),** *<0.001*	**4.99 (2.76–9.02),** *<0.001*	0.62 (0.39–1.01), *0.056*
IV antibiotics	**1.95 (1.41–2.70),** *<0.001*	**0.50 (0.36–0.70),** *<0.001*	**3.18 (2.17–4.67),** *<0.001*	2.33 (0.54–10.0), *0.255*	**1.76 (1.08–2.85),** *0.023*	**3.74 (1.15–12.1),** *0.028*	**2.33 (1.40–3.86),** *0.001*
Length of stay >48hours	**2.27 (1.58–3.25),** *<0.001*	1.40 (0.89–2.21), *0.145*	**3.76 (2.40–5.91),** *<0.001*	2.76 (0.36–21.2), *0.330*	1.65 (0.96–2.83), *0.068*	2.59 (0.79–8.53), *0.117*	2.10 (0.99–4.46), *0.052*
Length of stay≥6 days	**1.61 (1.25–2.06),** *<0.001*	**1.98 (1.48–2.64),** *<0.001*	**2.46 (1.91–3.18),** *<0.001*	**3.32 (1.35–8.13),** *0.009*	**1.61 (1.16–2.22),** *0.004*	**3.08 (1.70–5.57),** *<0.001*	**1.60 (1.06–2.42),** *0.025*
Length of stay≥12 days	**1.35 (1.01–1.80),** *0.042*	**2.94 (2.15–4.01),** *<0.001*	**2.59 (1.93–3.48),** *<0.001*	**3.08 (1.34–7.08),** *0.008*	**1.73 (1.22–2.47),** *0.002*	**3.53 (2.01–6.21),** *<0.001*	1.38 (0.87–2.18), *0.171*
Mortality	1.69 (0.99–2.87), *0.053*	**2.27 (1.34––3.86),** *0.002*	**3.74 (2.14–6.51),** *<0.001*	**4.68 (1.68–13.0),** *0.003*	**2.31 (1.31–4.07),** *0.004*	**3.14 (1.41–7.01),** *0.005*	1.43 (0.66–3.09), *0.367*
Severe outcomes (level2/3 admission or death)	**2.27 (1.63–3.16),** *<0.001*	**2.37 (1.69–3.31),** *<0.001*	**6.42 (4.49–9.18),** *<0.001*	**6.13 (2.64–14.2),** *<0.001*	**2.89 (2.01–4.16),** *<0.001*	**4.99 (2.82–8.81),** *<0.001*	**2.19 (1.39–3.46),** *0.001*

Values are unadjusted odds ratios (95% confidence intervals), significant values (p<0.05) in bold and p values in italic. X Not calculated as this criterion typically directs the outcome (use of supplemental oxygen).

Criterion A: In children and adults “Severe respiratory distress” was significantly associated with use of supplemental oxygen, use of mechanical ventilation, length of stay >48 hours & ≥6 days and severe outcome (level 2/3 admission and or death). In children “Severe respiratory distress” was also significantly associated with death, and in adults with use of intravenous antibiotics and length of stay ≥12 days.

Criterion B: In children and adults “Increased respiratory rate” was significantly associated with use of supplemental oxygen and length of stay (≥6 & ≥12 days). In adults “increased respiratory rate” was also significantly associated with use of mechanical ventilation. Increased respiratory rate was significantly associated with a decreased likelihood of intravenous antibiotic use in both children.

Criterion C: In children and adults “Peripheral oxygen saturation ≤92% breathing air, or on oxygen” was significantly associated with mechanical ventilation, intravenous antibiotic use, length of stay (>48 hours and ≥6 days) and severe outcome. In adults “Peripheral oxygen saturation ≤92% breathing air or on oxygen” was also significantly associated with length of stay (≥12 days) and death. Use of supplemental oxygen as an outcome measure was excluded *ad oculos* from this analysis, as peripheral oxygen saturation ≤92%, would with few exceptions trigger oxygen supplementation in adults and children. [5].

Criterion D: It was not possible to calculate odds ratios for “Respiratory exhaustion or apnoea” in children due to insufficient cases meeting this criterion. In adults “Respiratory exhaustion or apnoea” was significantly associated with use of supplemental oxygen, mechanical ventilation, increased length of stay (≥6 & ≥12 days), severe outcome and death.

Criterion E: In children and adults “Severe clinical dehydration or shock” was significantly associated with use of mechanical ventilation, severe outcome and death. In adults “Severe clinical dehydration or shock” was also significantly associated with use of supplemental oxygen, intravenous antibiotics and length of stay (≥6 & ≥12 days).

Criterion F: In children and adults “Altered conscious level” was significantly associated with use of supplemental oxygen, mechanical ventilation, severe outcome and death. In adults “altered conscious level” was also associated with intravenous antibiotic use and length of stay (≥6 & ≥12 days).

Criterion G: In children and adults the subjective criteria “Causing other clinical concern” was associated with intravenous antibiotic use and severe outcome. In children “causing other clinical concern” was also significantly associated with death, and in adults with length of stay (≥6 days). The most frequent examples of meeting this criterion included in children: fever alone or in combination with rash or mottled skin or pain or cough; and in adults: chest pain, fever alone or in combination with cough or pain.

**Figure 1:**
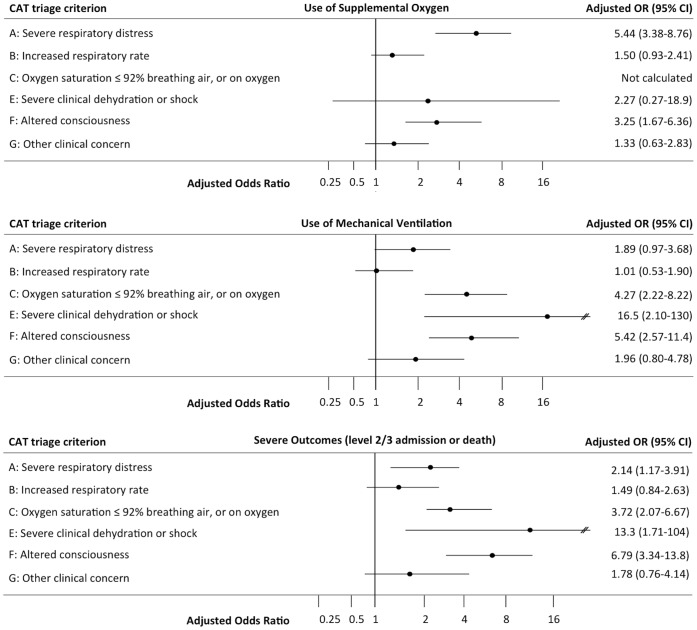
CAT criteria as independent predictors of use of oxygen, mechanical ventilation and severe outcomes in children. Multivariable analyses by forward stepwise regression of CAT criteria as independent predictors of use of oxygen [upper panel], mechanical ventilation [middle panel] and combined severe outcomes (level 2/3 admission or death)[lower panel] in children (<16 years).

**Figure 2:**
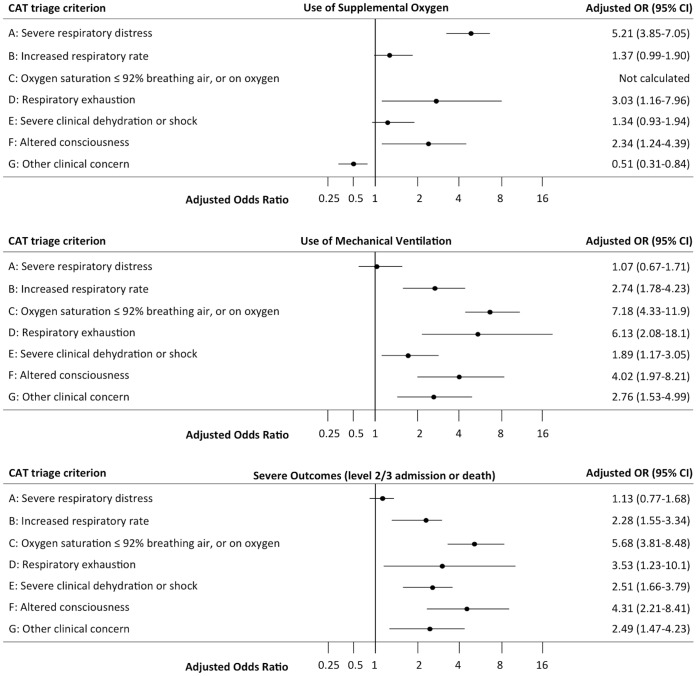
CAT criteria as independent predictors of use of oxygen, mechanical ventilation and severe outcomes in adults. Multivariable analyses by forward stepwise regression of CAT criteria as independent predictors of use of oxygen [upper panel], mechanical ventilation [middle panel] and combined severe outcomes (level 2/3 admission or death)[lower panel] in adults (≥16 years).

Summary results of multivariable analyses using the automatic stepwise forward selection procedure of CATs criteria at admission as independent predictors of outcomes (hospital-based intervention and patient outcomes) in children and adults are presented in Tables 4 & 5, and in Figures 1 & 2. Results were very similar when all criteria were retained in the model (Tables S2 & S3).

**Table 4:** Multivariable analyses of CAT criteria as independent predictors of outcomes in children (<16 years), forward stepwise regression.

CAT criteria	Supplementaloxygen	Mechanical ventilation	IV antibiotics	Length of stay>48 hours	Length of stay≥6 days	Length of stay≥12 days	Mortality	Severe outcomes (level2/3 admission or death)
A: Severe respiratorydistress	**5.44 (3.38–8.76),** *<0.001*	1.89 (0.97–3.68),*0.060*	1.23 (0.64–2.37),*0.538*	1.46 (0.84–2.54),*0.182*	1.30 (0.81–2.07),*0.274*	1.20 (0.69–2.07),*0.524*	2.58 (0.88–7.62),*0.085*	**2.14 (1.17–3.91),** *0.013*
B: Increasedrespiratory rate	1.50 (0.93–2.41),*0.094*	1.01 (0.53–1.90),*0.987*	**0.26 (0.15–0.43),** *<0.001*	1.16 (0.73–1.83),*0.536*	**2.01 (1.34–3.03),** *0.001*	**2.26 (1.40–3.63),** *0.001*	1.99 (0.70–5.65),*0.197*	1.49 (0.84–2.63),*0.171*
C: Oxygen saturation ≤92%	X	**4.27 (2.22–8.22),** *<0.001*	**4.40 (2.01–9.61),** *<0.001*	**3.84 (2.11–7.01),** *<0.001*	**1.66 (1.05–2.62),** *0.031*	0.95 (0.55–1.64),*0.852*	0.95 (0.31–2.92),*0.931*	**3.72 (2.07–6.67),** *<0.001*
D: Respiratory exhaustion	*	*	*	*	*	*	*	*
E: Severe clinical dehydration or shock	2.27 (0.27–18.90),*0.448*	**16.52 (2.10–130.14),** *0.008*	0.33 (0.03–3.28),*0.343*	0.55 (0.08–3.77),*0.544*	4.40 (0.70–27.5),*0.114*	2.49 (0.42–14.84),*0.318*	10.48 (0.90–122.77),*0.061*	**13.29 (1.71–103.6),** *0.013*
F: Alteredconsciousness	**3.25 (1.67–6.36),** *0.001*	**5.42 (2.57–11.42),** *<0.001*	0.99 (0.41–2.41),*0.981*	1.17 (0.56–2.42),*0.675*	1.13 (0.59–2.16),*0.718*	1.38 (0.67–2.83),*0.668*	2.26 (0.64–8.05),*0.207*	**6.79 (3.34–13.80),** *<0.001*
G: Other clinicalconcern	1.33 (0.63–2.82),*0.456*	1.96 (0.80–4.78),*0.139*	0.64 (0.26–1.53),*0.312*	1.08 (0.49–2.38),*0.855*	1.02 (0.49–2.09),*0.963*	1.21 (0.54–2.72),*0.636*	**4.24 (1.33–13.47),** *0.014*	1.78 (0.76–4.14),*0.183*

Values are adjusted odds ratios (95% confidence intervals), significant values in bold and p values in italic. Each predictor variable (CAT criterion) in model adjusted for other criteria in the multivariable model remaining following forward stepwise regression (significance level for addition to the model p≤0.05); * criteria excluded from the final model. X Not calculated as this criterion typically directs the outcome (use of supplemental oxygen).

**Table 5:** Multivariable analyses of CAT criteria as independent predictors of outcomes in adults (≥16 years), forward stepwise regression.

CAT criteria	Supplementaloxygen	Mechanicalventilation	IV antibiotics	Length of stay>48 hours	Length of stay≥6 days	Length of stay≥12 days	Mortality	Severe outcomes (level2/3 admission or death)
A: Severe respiratory distress	**5.21 (3.85–7.05),** *<0.001*	1.07 (0.67–1.71),*0.767*	**1.45 (1.01–2.09),** *0.041*	**1.59 (1.08–2.34),** *0.018*	1.17 (0.88–1.54),*0.275*	0.89 (0.64–1.25),*0.497*	0.98 (0.54–1.78),*0.952*	1.13 (0.77–1.68),*0.533*
B: Increasedrespiratory rate	1.37 (0.99–1.90),*0.059*	**2.74 (1.78–4.23),** *<0.001*	**0.44 (0.31–0.63),** *<0.001*	1.32 (0.82–2.10),*0.252*	**1.90 (1.41–2.57),** *<0.001*	**2.87 (2.07–3.96),** *<0.001*	**1.96 (1.12–3.41),** *0.018*	**2.28 (1.55–3.34),** *<0.001*
C: Oxygensaturation ≤92%	X	**7.18 (4.33–11.9),** *<0.001*	**2.80 (1.85–4.26),** *<0.001*	**3.06 (1.91–4.92),** *<0.001*	**2.16 (1.63–2.86),** *<0.001*	**2.46 (1.76–3.43),** *<0.001*	**3.18 (1.72–5.88),** *<0.001*	**5.68 (3.81–8.48),** *<0.001*
D: Respiratory exhaustion	**3.03 (1.16–7.96),** *0.024*	**6.13 (2.08–18.1),** *0.001*	1.36 (0.31–6.01),*0.689*	2.26 (0.28–18.1),*0.442*	2.16 (0.81–5.70),*0.122*	1.62 (0.61–4.29),*0.332*	2.00 (0.62–6.51),*0.248*	**3.53 (1.23–10.1),** *0.019*
E: Severe clinicaldehydration or shock	1.34 (0.93–1.94),*0.115*	**1.89 (1.17–3.05),** *0.009*	1.51 (0.91–2.50),*0.108*	1.42 (0.82–2.48),*0.211*	1.40 (0.99–1.97),*0.055*	**1.50 (1.03–2.20),** *0.036*	**1.82 (1.00–3.31),** *0.048*	**2.51 (1.66–3.79),** *<0.001*
F: Alteredconsciousness	**2.34 (1.24–4.39),** *0.008*	**4.02 (1.97–8.21),** *<0.001*	**3.34 (1.01–11.0),** *0.048*	2.42 (0.72–8.13),*0.154*	**2.61 (1.40–4.86),** *0.003*	**2.94 (1.60–5.39),** *0.001*	2.04 (0.84–4.94),*0.114*	**4.31 (2.21–8.41),** *<0.001*
G: Other clinicalconcern	**0.51 (0.31–0.84),** *0.009*	**2.76 (1.53–4.99),** *0.001*	**0.52 (0.31–0.88),** *0.014*	1.89 (0.88–4.07),*0.105*	**1.60 (1.04–2.48),** *0.033*	1.41 (0.86–2.31),*0.172*	1.31 (0.58–2.97),*0.515*	**2.49 (1.47–4.23),** *0.001*

Values are adjusted odds ratios (95% confidence intervals), significant values in bold and p values in italic. Each predictor variable (CAT criterion) in model adjusted for other criteria in the multivariable model remaining following forward stepwise regression (significance level for addition to the model p≤0.05); X Not calculated as this criterion typically directs the outcome (use of supplemental oxygen).

In children, use of supplemental oxygen was independently positively predicted by criteria A “Severe respiratory distress” and F “Altered conscious level, while association with criterion B “Increased respiratory rate” was weaker (Figure 1 upper panel). Use of mechanical ventilation was independently predicted by criteria C “Peripheral oxygen saturation ≤92% in air or being on oxygen”, E “Severe clinical dehydration or shock”, and F “Altered conscious level”, while association with criterion A “Severe respiratory distress” was weaker (Figure 1 middle panel). Decision to use intravenous antibiotics was predicted by criterion C “Peripheral oxygen saturation ≤92% on air or being on oxygen” but negatively predicted by criterion B “Increased respiratory rate” (Table 4). Length of stay >48 hours was independently predicted by criteria C. Length of stay ≥6 days was independently predicted by criteria B and C. Length of stay ≥12 days was independently predicted by criterion B (Table 4). Severe outcome (level 2/3 care or death) was predicted by criteria A “Severe respiratory distress”, C “Oxygen saturation ≤92%”, E “Severe clinical dehydration or shock” and F “Altered conscious level” (Figure 1 lower panel). Of note, mortality was only independently predicted in children by criteria G “Other clinical concern” [OR (95%CI); 4.24(1.33–13.47), p = 0.014] (Table 4).

In adults use of supplemental oxygen was independently positively predicted by criteria A “Severe respiratory distress”, B “Increased respiratory rate”, D “Respiratory exhaustion”, F “Altered conscious level” and negatively by G “Other clinical concern” (Figure 2 upper panel). Use of mechanical ventilation was independently positively predicted by criteria B “Increased respiratory rate”, C “Oxygen saturation ≤92%”, D “Respiratory exhaustion”, E “Severe dehydration or shock”, F “Altered conscious level” and G “Other clinical concern” (Figure 2 middle panel). Intravenous antibiotic use was independently predicted by criteria A, C, and F, but negatively predicted by criteria B and G (Table 5). Length of stay >48 hours was independently predicted by criteria A and C (Table 5). Length of stay ≥6 days was independently predicted by criteria B, C, F and G. Length of stay ≥12 days was independently predicted by criteria B, C, E and F. Severe outcome was independently predicted by criteria B “Increased respiratory rate”, “Oxygen saturation ≤92%”, D “Respiratory exhaustion”, E “Severe clinical dehydration or shock”, F “Altered consciousness” and G “Other clinical concern” (Figure 2 lower panel). Mortality was independently predicted by criteria B “Increased respiratory rate”, C “Oxygen saturation ≤92%”, and E “Severe clinical dehydration or shock” (Table 5).

## Discussion

Hospital capacity and resources are strictly limited during a pandemic. Each of the CATs criteria had an independent role in identifying both use of clinical interventions that would in normal circumstances only be provided in hospital and adverse patient outcome measures, suggesting that had a “surge” threshold been reached in the UK during the 2009/10 pandemic, these triage tools would have been fit for purpose. Based on multivariable analyses none of the criteria were redundant. This is reassuring given that criteria A to F were selected for proven performance in identifying severe illness in previous well regarded studies and all criteria are recognised in current national and international guidance. [5], [8]–[17] This also suggests that the potential exists for creating a simple unweighted scoring system similar to CURB-65 or PMEWS using the CATs criteria. A direct comparison of the overall performance (whole tool rather than individual criteria) of CATs, CURB-65 and PMEWS as triage tools using the same cohort data set is the subject of a related paper. [22] CATs were, if anything, slightly better performing than CURB-65 and PMEWS during the first two waves of the 2009/10 pandemic in the UK.

Criteria C – “Peripheral oxygen saturation ≤92% breathing air, or on oxygen” performed well (in terms of magnitude of odds ratio and statistical power) in predicting use of resources and outcomes across all analyses in both adults and children. Hypoxaemia is particularly difficult to assess in children by clinical observation. Our findings support the routine use of age-appropriate peripheral oxygen saturation measurement in assessing severity of disease in patients of all ages. Given the relatively low cost of such monitoring equipment, this observation may be particularly pertinent in terms of resource poor settings in other countries.

Criterion G – “Causing other clinical concern to their own GP or clinical team” was developed by a workgroup of experts in primary care and emergency care. In consensus they insisted on including a subjective criterion that allowed “a clinician in primary care to uses their own judgement to refer a patient to hospital” and “a clinician in hospital to use their judgement to admit a patient” in cases where other criteria may not have been met. It might be expected that very many patients would meet this subjective criterion and that the criterion would have poor discriminatory power and no validity but quite the opposite was observed. In multivariate analysis criterion G was the only criteria that independently predicted death in children. In adults criterion G independently predicted length of stay ≥6 days, mechanical ventilation and combined severe outcome (level 2/3 admission or death). Meeting this criterion in children was often based on recognition of fever plus another sign, and in adults recognition of chest pain or fever plus another sign. This finding supports the role of clinical judgment, be it founded on acumen, experience or instinctive “gut feeling”, as a important independent predictor of serious illness in people of all ages. [18].

### Limitations

CATs were being developed in preparation for a severe pandemic event when the A(H1N1)2009 influenza virus emerged. As a consequence the value of the individual criteria and overall validity of CATs (content validity, reliability, measurement properties and ability to detect change) were not tested in a prospective study as would be expected in an ideal situation. [23] Instead the CATs were rapidly finalised and made ready for use in the event of surge. Here we have presented an evaluation of the potential role of CATs criteria to predict interventions and outcomes in a pragmatic study conducted during a pandemic using the extensive data provided by the prospective multicentre FLU-CIN cohort. By design it was not possible to assess intra-observer agreement, inter-observer agreement or ability to detect change.

A potential limitation of this study relates to possible misclassification of criteria. This could happen because of an error in assessment of the patient by the clinician or because of a failure to check whether a criterion was actually ‘not present’. This is an inherent bias in all observational studies using routine clinical data and there is no way to verify this. However any bias caused by misclassification of criteria (the independent predictor variable) as not being present when in reality that criterion is present, will minimise differences in comparisons and so where we describe significant differences, these are likely to be robust.

### Generalisability

This study is limited to cases of proven A(H1N1)pdm09 influenza requiring hospital admission. It should be noted that it was not the aim of this study to derive a clinical predictor tool. CATs had already been proposed using a conceptual clinical framework but there were no baseline data on performance in a similar pandemic situation to enable meaningful validation in the FLU-CIN surveillance cohort. The aim of this study was, therefore, to explore the potential applicability of CATs as a clinical triage tool and contribute to its development in terms of whether the initial proposal to treat each criterion equally as a stand-alone trigger was justified. Further work is needed to validate CATs in an external cohort which could be drawn from other settings during the previous pandemic period or by means of prospective validation in future pandemic or epidemic scenarios and to consider using a combined or weighted scoring strategy. Moreover, it is recognised that the predictive performance of tools tested in secondary care settings may not be generalisable to primary care settings. Therefore the next step will be an assessment of the performance of CATs and other triage tools in the community.

Morbidity and mortality rates were low during the 2009/10 pandemic when compared to some previous influenza pandemics. Accordingly, the discriminatory capabilities of CATs might well differ where a pathogen has greater virulence.

The CATs were developed for use during pandemic events and their criteria address the most likely modes of critical illness arising from influenza, or the complications of influenza. CATs were also designed to identify sick patients most likely to benefit from higher levels of care due to other illnesses, which at presentation are indistinguishable from influenza like illness. CATs may have value in other scenarios where a high triage threshold is required for both adults and children such as other severe acute respiratory pandemic events, and possibly in some sudden impact health emergencies producing mass casualties.

## Conclusions

This study shows that CATs are potentially useful predictors of both use of hospital-based interventions and severe patient outcomes during an influenza pandemic. Each of the CATs criteria had a role in predicting a given outcome and none are redundant. It is also notable and novel that both paediatric and adults CATs were developed upon a common framework of assessment. Importantly this confers an element of equity in a situation where children and adults may have to compete for access to a limited common resource such as mechanical ventilators.

The CATs presented in this paper represent the first attempt to provide frontline healthcare professionals working across a national health service in communities and hospitals with high threshold triage criteria that could help make consistent, albeit difficult, decisions during extreme situations when health care demand exceeds capacity.

## Supporting Information

Table S1 **Distribution of subjects by criterion for each outcome measure (children and adults).**
(DOCX)

Table S2 **Summary results of multivariable analyses of CAT criteria as independent predictors of outcomes in children (<16 years).**
(DOCX)

Table S3 **Summary results of multivariable analyses of CAT criteria as independent predictors of outcomes in adults (≥16 years).**
(DOCX)

Appendix S1 **Paediatric Community Assessment Tool.**
(DOCX)

Appendix S2 **Adult Community Assessment Tool.**
(DOCX)

Appendix S3 **Alder Hey Children’s Hospital NHS Foundation Trust Pandemic Influenza Group.**
(DOCX)
