# Supplementary material for: An Evaluation of Community Assessment Tools (CATs) in Predicting Use of Clinical Interventions and Severe Outcomes during the A(H1N1)pdm09 Pandemic
Source: PLoS One. 2013 Sep 19;8(9):e75384. doi: 10.1371/journal.pone.0075384 (PMC3777884; doi:10.1371/journal.pone.0075384)
Supplement: Table S1 — Distribution of subjects by criterion for each outcome measure (children and adults). (DOCX) [file pone.0075384.s001.docx]

Supplementary Tables S1. Distribution of subjects (children and adults) by criterion for each outcome measure

Criterion A: Severe respiratory distress

| **Outcome measure** | **Criterion A present n (%)** | | |
| --- | --- | --- | --- |
|  | **Children (<16 yrs)**  **(n=480)** | **Adults**  **(n=1040)** | **All ages**  **(n=1520)** |
| Length of stay >48 hours  No  Yes | 23 (17.29)  110 (82.71) | 54 (10.21)  475 (89.79) | 77 (11.63)  583 (88.37) |
| Length of stay ≥6 days  No  Yes | 81 (60.90)  52 (39.10) | 280 (52.93)  249 (47.07) | 361 (54.53)  301 (45.47) |
| Length of stay ≥12 days  No  Yes | 104 (78.20)  29 (21.80) | 391 (73.91)  138 (26.09) | 495 (74.77)  167 (25.23) |
| Additional oxygen requirement  No  Yes | 72 (54.14)  61 (45.86) | 280 (52.93)  249 (47.07) | 352 (53.17)  310 (46.83) |
|  |  |  |  |
| Need for mechanical ventilation  No  Yes | 105 (78.95)  28 (21.05) | 439 (82.99)  90 (17.01) | 544 (82.18)  118 (17.82) |
|  |  |  |  |
| In-hospital antibiotics  No  Yes | 17 (12.78)  116 (87.22) | 70 (13.23)  459 (86.77) | 87 (13.14)  575 (86.86) |
|  |  |  |  |
| Mortality  No  Yes | 123 (92.48)  10 (7.52) | 490 (92.63)  39 (7.37) | 613 (92.60)  49 (7.40) |
|  |  |  |  |
| Adverse outcome (level 2/3 admission or death)  No  Yes | 95 (71.43)  38 (28.57) | 401 (75.80)  128 (24.20) | 496 (74.92)  166 (25.08) |
|  |  |  |  |

Criterion B: Increased respiratory rate

| **Outcome measure** | **Criterion B present n (%)** | | |
| --- | --- | --- | --- |
|  | **Children (<16 yrs)**  **(n=480)** | **Adults**  **(n=1040)** | **All ages**  **(n=1520)** |
| Length of stay >48 hours  No  Yes | 40 (21.86)  143 (78.14) | 26 (10.44)  223 (89.56) | 66 (15.28)  366 (84.72) |
|  |  |  |  |
| Length of stay ≥6 days  No  Yes | 109 (59.56)  74 (40.44) | 114 (45.78)  135 (54.22) | 223 (51.6)  209 (48.38) |
| Length of stay ≥12 days  No  Yes | 135 (73.77)  48 (26.23) | 150 (60.24)  99 (39.76) | 285 (65.97)  147 (34.03) |
| Additional oxygen requirement  No  Yes | 132 (72.13)  51 (27.87) | 155 (62.25)  94 (37.75) | 287 (66.44)  145 (33.56) |
|  |  |  |  |
| Need for mechanical ventilation  No  Yes | 160 (87.43)  23 (12.57) | 192 (77.11)  57 (22.89) | 352 (81.48)  80 (18.52) |
|  |  |  |  |
| In-hospital antibiotics  No  Yes | 48 (26.23)  135 (73.77) | 66 (26.51)  183 (73.49) | 114 (26.39)  318 (73.61) |
|  |  |  |  |
| Mortality  No  Yes | 173 (94.54)  10 (5.46) | 224 (89.96)  25 (10.04) | 397 (91.90)  35 (8.10) |
|  |  |  |  |
| Adverse outcome (level 2/3 admission or death)  No  Yes | 147 (80.33)  36 (19.67) | 176 (70.68)  73 (29.32) | 323 (74.77)  109 (25.23) |
|  |  |  |  |

Criterion C: Oxygen saturation ≤92% in air or on oxygen

| **Outcome measure** | **Criterion C present n (%)** | | |
| --- | --- | --- | --- |
|  | **Children (<16 yrs)**  **(n=480)** | **Adults**  **(n=1040)** | **All ages**  **(n=1520)** |
| Length of stay >48 hours  No  Yes | 16 (11.51)  123 (88.49) | 25 (6.07)  387 (93.93) | 41 (7.44)  510 (92.56) |
|  |  |  |  |
| Length of stay ≥6 days  No  Yes | 82 (58.99)  57 (41.01) | 187 (45.39)  225 (54.61) | 269 (48.82)  282 (51.18) |
| Length of stay ≥12 days  No  Yes | 111 (79.86)  28 (20.14) | *272 (66.02)*  *140 (33.98)* | 383 (69.51)  168 (30.49) |
| Need for mechanical ventilation  No  Yes | 102 (73.38)  37 (26.62) | 307 (74.51)  105 (25.49) | 409 (74.23)  142 (25.77) |
|  |  |  |  |
| In-hospital antibiotics  No  Yes | 9 (6.47)  130 (93.53) | 37 (8.98)  375 (91.02) | 46 (8.35)  505 (91.65) |
|  |  |  |  |
| Mortality  No  Yes | 132 (94.96)  7 (5.04) | 369 (89.56)  43 (10.44) | 501 (90.93)  50 (9.07) |
|  |  |  |  |
| Adverse outcome (level 2/3 admission or death)  No  Yes | 93 (66.91)  46 (33.09) | 269 (65.29)  143 (34.71) | 362 (65.70)  189 (34.30) |
|  |  |  |  |

Criterion D: Respiratory exhaustion or apnoeic episode

| **Outcome measure** | **Criterion D present n (%)** | | |
| --- | --- | --- | --- |
|  | **Children (<16 yrs)**  **(n=480)** | **Adults**  **(n=1040)** | **All ages**  **(n=1520)** |
| Length of stay >48 hours  No  Yes | 0 (0.00)  1 (100.00) | 1 (4.35)  22 (95.65) | 1 (4.17)  23 (95.83) |
|  |  |  |  |
| Length of stay ≥6 days  No  Yes | 0 (0.00)  1 (100.00) | 7 (30.43)  16 (69.57) | 7 (29.17)  17 (70.83) |
| Length of stay ≥12 days  No  Yes | 0 (0.00)  1 (100.00) | 12 (52.17)  11 (47.83) | 12 (50.00)  12 (50.00) |
| Additional oxygen requirement  No  Yes | 1 (100.00)  0 (0.00) | 8 (34.78)  15 (65.22) | 9 (37.50)  15 (62.50) |
|  |  |  |  |
| Need for mechanical ventilation  No  Yes | 1 (100.00)  0 (0.00) | 10 (43.48)  13 (56.52) | 11 (45.83)  13 (54.17) |
|  |  |  |  |
| In-hospital antibiotics  No  Yes | 1 (100.00)  0 (0.00) | 2 (8.70)  21 (91.30) | 3 (12.50)  21 (87.50) |
|  |  |  |  |
| Mortality  No  Yes | 0 (0.00)  1 (100.00) | 18 (78.26)  5 (21.74) | 18 (75.00)  6 (25.00) |
|  |  |  |  |
| Adverse outcome (level 2/3 admission or death)  No  Yes | 0 (0.00)  1 (100.00) | 10 (43.48)  13 (56.52) | 10 (41.67)  14 (58.33) |
|  |  |  |  |

Criterion E: Severe clinical dehydration or shock

| **Outcome measure** | **Criterion E present n (%)** | | |
| --- | --- | --- | --- |
|  | **Children (<16 yrs)**  **(n=480)** | **Adults**  **(n=1040)** | **All ages**  **(n=1520)** |
| Length of stay >48 hours  No  Yes | 2 (33.33)  4 (66.67) | 17 (9.66)  159 (90.34) | 19 (10.44)  163 (89.56) |
|  |  |  |  |
| Length of stay ≥6 days  No  Yes | 2 (33.33)  4 (66.67) | 86 (48.86)  90 (51.14) | 88 (48.35)  94 (51.65) |
| Length of stay ≥12 days  No  Yes | 4 (66.67)  2 (33.33) | 119 (67.61)  57 (32.39) | 123 (67.58)  59 (32.42) |
| Additional oxygen requirement  No  Yes | 4 (66.67)  2 (33.33) | 108 (61.36)  68 (38.64) | 112 (61.54)  70 (38.46) |
|  |  |  |  |
| Need for mechanical ventilation  No  Yes | 3 (50.00)  3 (50.00) | 137 (77.84)  39 (22.16) | 140 (76.92)  42 (23.08) |
|  |  |  |  |
| In-hospital antibiotics  No  Yes | 2 (33.33)  4 (66.67) | 21 (11.93)  155 (88.07) | 23 (12.64)  159 (87.36) |
|  |  |  |  |
| Mortality  No  Yes | 4 (66.67)  2 (33.33) | 157 (89.20)  19 (10.80) | 161 (88.46)  21 (11.54) |
|  |  |  |  |
| Adverse outcome (level 2/3 admission or death)  No  Yes | 2 (33.33)  4 (66.67) | 116 (65.91)  60 (34.09) | 118 (64.84)  64 (35.16) |
|  |  |  |  |

Criterion F: Altered consciousness

| **Outcome measure** | **Criterion F present n (%)** | | |
| --- | --- | --- | --- |
|  | **Children (<16 yrs)**  **(n=480)** | **Adults**  **(n=1040)** | **All ages**  **(n=1520)** |
| Length of stay >48 hours  No  Yes | 12 (22.64)  41 (77.36) | 3 (5.77)  49 (94.23) | 15 (14.29)  90 (85.71) |
|  |  |  |  |
| Length of stay ≥6 days  No  Yes | 35 (66.04)  18 (33.96) | 17 (32.69)  35 (67.31) | 52 (49.52)  53 (50.48) |
| Length of stay ≥12 days  No  Yes | 41 (77.36)  12 (22.64) | 26 (50.00)  26 (50.00) | 67 (63.81)  38 (36.19) |
| Additional oxygen requirement  No  Yes | 32 (60.38)  21 (39.62) | 26 (50.00)  26 (50.00) | 58 (55.24)  47 (44.76) |
|  |  |  |  |
| Need for mechanical ventilation  No  Yes | 34 (64.15)  19 (35.85) | 32 (61.54)  20 (38.46) | 66 (62.86)  39 (37.14) |
|  |  |  |  |
| In-hospital antibiotics  No  Yes | 8 (15.09)  45 (84.91) | 3 (5.77)  49 (94.23) | 11 (10.48)  94 (89.52) |
|  |  |  |  |
| Mortality  No  Yes | 48 (90.57)  5 (9.43) | 44 (84.62)  8 (15.38) | 92 (87.62)  13 (12.38) |
|  |  |  |  |
| Adverse outcome (level 2/3 admission or death)  No  Yes | 28 (52.83)  25 (47.17) | 26 (50.00)  26 (50.00) | 54 (51.43)  51 (48.57) |
|  |  |  |  |

Criterion G: Other clinical concern

| **Outcome measure** | **Criterion G present n (%)** | | |
| --- | --- | --- | --- |
|  | **Children (<16 yrs)**  **(n=480)** | **Adults**  **(n=1040)** | **All ages**  **(n=1520)** |
| Length of stay >48 hours  No  Yes | 10 (24.39)  31 (75.61) | 8 (8.00)  92 (92.00) | 18 (12.77)  123 (87.23) |
|  |  |  |  |
| Length of stay ≥6 days  No  Yes | 28 (68.29)  13 (31.71) | 48 (48.00)  52 (52.00) | 76 (53.90)  65 (46.10) |
| Length of stay ≥12 days  No  Yes | 32 (78.05)  9 (21.95) | 71 (71.00)  29 (29.00) | 103 (73.05)  38 (26.95) |
| Additional oxygen requirement  No  Yes | 27 (65.85)  14 (34.15) | 75 (75.00)  25 (25.00) | 102 (72.34)  39 (27.66) |
|  |  |  |  |
| In-hospital antibiotics  No  Yes | 8 (19.51)  33 (80.49) | 25 (25.00)  75 (75.00) | 33 (23.40)  108 (76.60) |
|  |  |  |  |
| Mechanical ventilation  No  Yes | 31 (75.61)  10 (24.39) | 77 (77.00)  23 (23.00) | 108 (76.60)  33 (23.40) |
| Mortality  No  Yes | 36 (87.80)  5 (12.20) | 92 (92.00)  8 (8.00) | 128 (90.78)  13 (9.22) |
|  |  |  |  |
| Adverse outcome (level 2/3 admission or death)  No  Yes | 29 (70.73)  12 (29.27) | 69 (69.00)  31 (31.00) | 98 (69.50)  43 (30.50) |
