# Supplementary material for: An Evaluation of Community Assessment Tools (CATs) in Predicting Use of Clinical Interventions and Severe Outcomes during the A(H1N1)pdm09 Pandemic
Source: PLoS One. 2013 Sep 19;8(9):e75384. doi: 10.1371/journal.pone.0075384 (PMC3777884; doi:10.1371/journal.pone.0075384)
Supplement: Table S2 — Summary results of multivariable analyses of CAT criteria as independent predictors of outcomes in children (<16 years). (DOCX) [file pone.0075384.s002.docx]

Supplementary Table S2. Summary results of multivariable analyses of CAT criteria as independent predictors of outcomes in children (< 16 years)

| CAT criteria | Supplemental oxygen | Mechanical ventilation | IV antibiotics | Length of stay >48 hours | Length of stay ≥6 days | Length of stay ≥12 days | Mortality | Severe outcomes (level 2/3 admission or death) |
| --- | --- | --- | --- | --- | --- | --- | --- | --- |
| A: severe respiratory distress | **5.44 (3.38-8.76),** *<0.001* | 1.89 (0.97-3.68), *0.060* | 1.23 (0.64-2.37), *0.538* | 1.48 (0.86-2.55), *0.156* | 1.30 (0.81-2.07), *0.274* | 1.17 (0.67-2.02), *0.585* | 2.58 (0.88-7.62), *0.085* | **2.14 (1.17-3.91),** *0.013* |
| B: increased respiratory rate | 1.50 (0.93-2.41), *0.094* | 1.01 (0.53-1.90), *0.987* | **0.26 (0.15-0.43),** *<0.001* | 1.42 (0.91-2.21), *0.125* | **2.01 (1.34-3.03),** *0.001* | **2.19 (1.36-3.54),** *0.001* | 1.99 (0.70-5.65), *0.197* | 1.49 (0.84-2.63), *0.171* |
| C: oxygen saturation ≤ 92% | X | **4.27 (2.22-8.22),** *<0.001* | **4.40 (2.01-9.61),** *<0.001* | **3.40 (1.88-6.17),** *<0.001* | **1.66 (1.05-2.62),** *0.031* | 0.98 (0.56-1.69), *0.933* | 0.95 (0.31-2.92), *0.931* | **3.72 (2.07-6.67),** *<0.001* |
| D: respiratory exhaustion | * | * | * | * | * | * | * | * |
| E: severe clinical dehydration or shock | 2.27 (0.27-18.90), *0.448* | **16.52 (2.10-130.14),** *0.008* | 0.33 (0.03-3.28), *0.343* | 0.51 (0.08-3.46), *0.493* | 4.40 (0.70-27.52), *0.114* | 1.53 (0.17-14.24), *0.706* | 10.48 (0.90-122.77), *0.061* | **13.29 (1.71-103.55),** *0.013* |
| F: altered consciousness | **3.25 (1.67-6.36),** *0.001* | **5.42 (2.57-11.42),** *<0.001* | 0.99 (0.41-2.41), *0.981* | 1.19 (0.58-2.42), *0.635* | 1.13 (0.59-2.16), *0.718* | 1.30 (0.62-2.72), *0.481* | 2.26 (0.64-8.05), *0.207* | **6.79 (3.34-13.80),** *<0.001* |
| G: other clinical concern | 1.33 (0.63-2.82), *0.456* | 1.96 (0.80-4.78), *0.139* | 0.64 (0.26-1.53), *0.312* | 1.06 (0.49-2.32), *0.875* | 1.02 (0.49-2.09), *0.963* | 1.23 (0.55-2.74), *0.619* | **4.24 (1.33-13.47),** *0.014* | 1.78 (0.76-4.14), *0.183* |

Values are adjusted odds ratios (95% confidence intervals), significant values (p≤0.05) in bold and p values in italic. Each predictor variable (CAT criterion) in model adjusted for each other. *OR could not be calculated because of insufficient data. X Not calculated as this criterion usually directs the outcome (use of supplemental oxygen).
