# Supplementary material for: An Evaluation of Community Assessment Tools (CATs) in Predicting Use of Clinical Interventions and Severe Outcomes during the A(H1N1)pdm09 Pandemic
Source: PLoS One. 2013 Sep 19;8(9):e75384. doi: 10.1371/journal.pone.0075384 (PMC3777884; doi:10.1371/journal.pone.0075384)
Supplement: Table S3 — Summary results of multivariable analyses of CAT criteria as independent predictors of outcomes in adults (≥16 years). (DOCX) [file pone.0075384.s003.docx]

Supplementary Table S3. Summary results of multivariable analyses of CAT criteria as independent predictors of outcomes in adults (≥ 16 years)

| CAT criteria | Supplemental oxygen | Mechanical ventilation | IV antibiotics | Length of stay >48 hours | Length of stay ≥6 days | Length of stay ≥12 days | Mortality | Severe outcomes (level 2/3 admission or death) |
| --- | --- | --- | --- | --- | --- | --- | --- | --- |
| A: severe respiratory distress | **5.21 (3.85-7.05),** *<0.001* | 1.07 (0.67-1.71), *0.767* | **1.45 (1.01-2.09),** *0.041* | **1.47 (1.00-2.15),** *0.049* | 1.17 (0.88-1.54), *0.275* | 0.89 (0.64-1.24), *0.497* | 0.98 (0.54-1.78), *0.952* | 1.13 (0.77-1.68), *0.533* |
| B: increased respiratory rate | 1.37 (0.99-1.90), *0.059* | **2.74 (1.78-4.23),** *<0.001* | **0.44 (0.31-0.63),** *<0.001* | **1.64 (1.03-2.60),** *0.035* | **1.90 (1.41-2.57),** *<0.001* | **2.87 (2.07-3.96),** *<0.001* | **1.96 (1.12-3.41),** *0.018* | **2.28 (1.55-3.34),** *<0.001* |
| C: oxygen saturation ≤ 92% | X | **7.18 (4.33-11.91),** *<0.001* | **2.80 (1.85-4.26),** *<0.001* | **3.32 (2.08-5.33),** *<0.001* | **2.16 (1.63-2.86),** *<0.001* | **2.46 (1.76-3.43),** *<0.001* | **3.18 (1.72-5.88),** *<0.001* | **5.68 (3.81-8.48),** *<0.001* |
| D: respiratory exhaustion | **3.03 (1.16-7.96),** *0.024* | **6.13 (2.08-18.07),** *0.001* | 1.36 (0.31-6.01), *0.689* | 2.44 (0.31-19.11), *0.394* | 2.16 (0.81-5.70), *0.122* | 1.62 (0.61-4.29), *0.332* | 2.00 (0.62-6.51), *0.248* | **3.53 (1.23-10.08),** *0.019* |
| E: Severe clinical dehydration or shock | 1.34 (0.93-1.94), *0.115* | **1.89 (1.17-3.05),** *0.009* | 1.51 (0.91-2.50), *0.108* | 1.51 (0.87-2.61), *0.141* | 1.40 (0.99-1.97), *0.055* | **1.50 (1.03-2.20),** *0.036* | **1.82 (1.00-3.31),** *0.048* | **2.51 (1.66-3.79),** *<0.001* |
| F: altered consciousness | **2.34 (1.24-4.39),** *0.008* | **4.02 (1.97-8.21),** *<0.001* | **3.34 (1.01-11.03),** *0.048* | 2.44 (0.73-8.13), *0.146* | **2.61 (1.40-4.86),** *0.003* | **2.94 (1.60-5.39),** *0.001* | 2.04 (0.84-4.94), *0.114* | **4.31 (2.21-8.41),** *<0.001* |
| G: other clinical concern | **0.51 (0.31-0.84),** *0.009* | **2.76 (1.53-4.99),** *0.001* | **0.52 (0.31-0.88),** *0.014* | 2.03 (0.95-4.35), *0.067* | **1.60 (1.04-2.48),** *0.033* | 1.41 (0.86-2.31), *0.172* | 1.31 (0.58-2.97), *0.515* | **2.49 (1.47-4.23),** *0.001* |

Values are adjusted odds ratios (95% confidence intervals), significant values (p≤0.05) in bold and p values in italic. Each predictor variable (CAT criterion) in model adjusted for each other. X Not calculated as this criterion usually directs the outcome (use of supplemental oxygen).
