# Supplementary figures and images for: An Evaluation of Community Assessment Tools (CATs) in Predicting Use of Clinical Interventions and Severe Outcomes during the A(H1N1)pdm09 Pandemic
Source: PLoS One. 2013 Sep 19;8(9):e75384. doi: 10.1371/journal.pone.0075384 (PMC3777884; doi:10.1371/journal.pone.0075384)

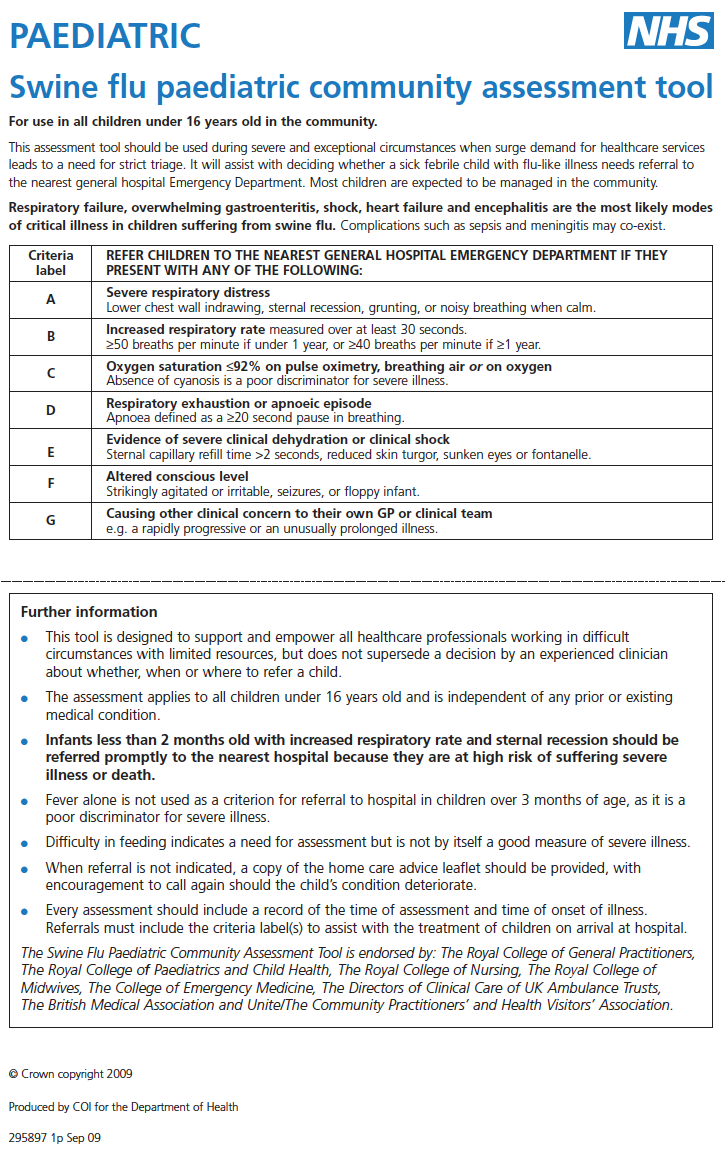

Supplement: Appendix S1 — Paediatric Community Assessment Tool. (DOCX) [file pone.0075384.s004.docx]

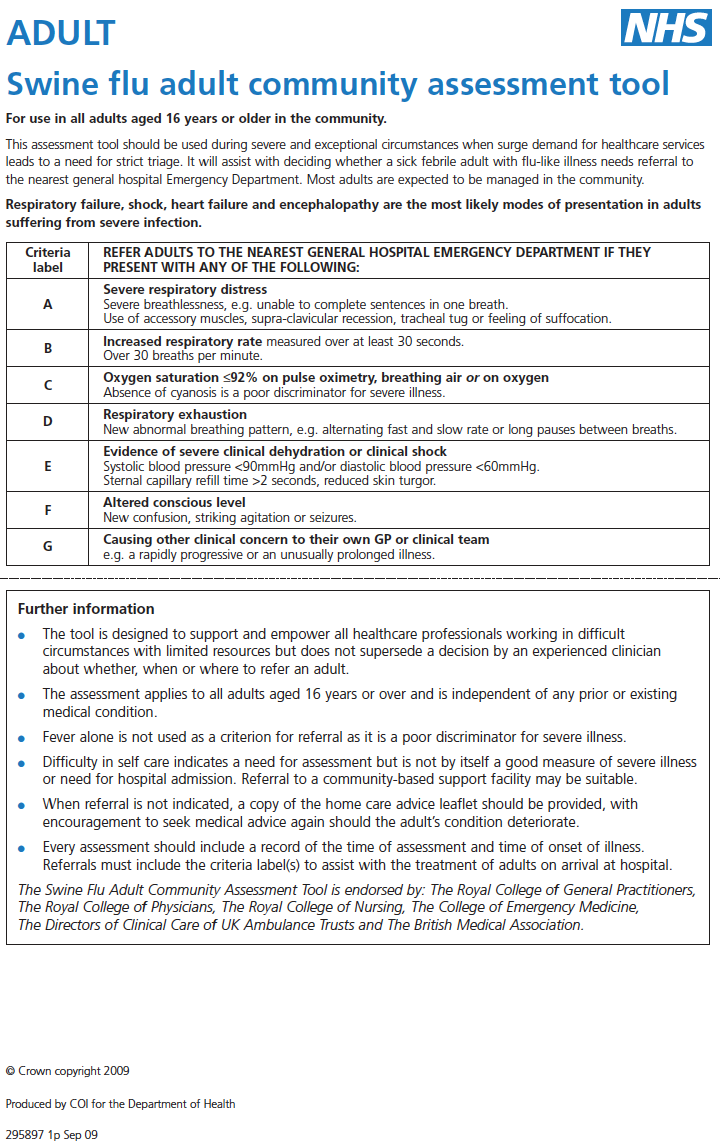

Supplement: Appendix S2 — Adult Community Assessment Tool. (DOCX) [file pone.0075384.s005.docx]
