# Supplementary material for: An Evaluation of Community Assessment Tools (CATs) in Predicting Use of Clinical Interventions and Severe Outcomes during the A(H1N1)pdm09 Pandemic
Source: PLoS One. 2013 Sep 19;8(9):e75384. doi: 10.1371/journal.pone.0075384 (PMC3777884; doi:10.1371/journal.pone.0075384)
Supplement: Appendix S3 — Alder Hey Children’s Hospital NHS Foundation Trust Pandemic Influenza Group. (DOCX) [file pone.0075384.s006.docx]

**Alder Hey Children’s Hospital NHS Foundation Trust Pandemic Influenza Group.**

Strategy Group:
Malcolm G Semple (Convenor), Louisa Heaf, Pauline Holt, Steve Kerr, Sharon Lowe, Ravi Massey, James Mwenechanya, Andrew Riordan, Eve Stemson, Joanne Noblet, Dianne Topping, Elvina White, Pete Arrowsmith, and Tony Rigby.

Internal Contributors and reviewers:
Paul Baines, Lynda Brook, Nigel Cunliffe, Diane Dean, Robert Johnson, Rachel Kneen, Ildiko Kustos, Tony Nunn, Catrin Barker.

External Contributors and reviewers:
Frank Whitford, Ass. Director of Health Service Resilience, NHS North West; Chris Bedford, Consultant Paediatrician, Warrington & Halton Hospitals NHS Foundation Trust; Marc England & Partners at The Whitby Group Practice, Western Cheshire PCT; Jo Hardie, & Partners, The Group Practice Surgery, Western Cheshire PCT; Patrick Boyd & Partners, Roseneath Medical Practice, Flintshire LHB; Nicholas Prigg & Partners, Chirk Surgery, **Betsi Cadwaladr University LHB**; Liverpool Central PCT Board and Sefton PCT Board.
